# Effect of Melatonin and TNF-α on Differentiation of PDL Stem Cells

**DOI:** 10.2174/0109298673412297260520021501

**Published:** 2026-07-09

**Authors:** Ting Liu, Liuyun Zheng, Dinesh Rokaya, Hao-Ying Li, Yang Cai

**Affiliations:** 1 Department of Stomatology, Guizhou Medical University, Guiyang, 550004, China;; 2 Department of Stomatology, School of Medicine, Guangzhou First People's Hospital, South China University of Technology, Guangzhou, China;; 3 Clinical Sciences Department, College of Dentistry, Ajman University, Ajman, United Arab Emirates;; 4 Center of Medical and Bio-Allied Health Sciences Research, Ajman University, United Arab Emirates;; 5 Institute of Pharmaceutical Science, King’s College London, LondonSE1 9NH, United Kingdom

**Keywords:** Melatonin, periodontitis, human periodontal ligament stem cells, osteogenesis, osteogenic differentiation, oxidative stress, TNF-α, alveolar bone regeneration

## Abstract

**Introduction:**

Melatonin (MEL) has anti-inflammatory, antioxidant, and immune regulatory properties. This study aimed to evaluate MEL as an antioxidant in the osteogenesis process of the human periodontal ligament stem cells (hPDLSCs).

**Methods:**

This is an *in vitro* experimental laboratory study using human periodontal ligament stem cells (hPDLSCs). were cultured and characterized *in vitro*. TNF-α was administered at 10 ng/ml. The ALP activity of hPDLSCs and the expressions of two genes, Runx2 and OPN, were also studied. ALP staining and activity assays were conducted on day 7 and day 14 to study the effects of TNF-α and MEL on bone formation.

**Results:**

It showed that MEL counteracted the ROS generation and the inhibitory osteogenesis of hPDLSCs induced by TNF-α, by upregulating the expression of antioxidases and downregulating the expression of oxidases. TNF-α treatment resulted in a decrease in ALP staining and activity, and MEL was able to result in TNF-α-induced impaired osteogenesis of hPDLSCs. Similarly, MEL inhibited the phosphorylation of p65 protein and blocked the degradation of IκBα protein.

**Discussion:**

This study demonstrates that melatonin preserves the osteogenic potential of human periodontal ligament stem cells under TNF-α-induced inflammatory conditions. By reducing ROS, enhancing antioxidant defenses, and suppressing oxidases, melatonin restores ALP activity and increases Runx2 and OPN expression. These effects occur through inhibition of NF-κB signaling, indicating its protective role in inflammatory microenvironments.

**Conclusion:**

MEL effectively attenuates TNF-α-induced oxidative stress and restores the osteogenic differentiation capacity of human periodontal ligament stem cells in an inflammatory microenvironment. Its antioxidant and anti-inflammatory properties support its potential use in periodontal regeneration therapies.

## 1 INTRODUCTION

Periodontitis is caused by long-term bacterial infection of the periodontal support tissue and can cause damage to periodontal tissue, leading to a gradual periodontal attachment loss and alveolar bone resorption, which results in loss of tooth. Periodontitis is a common cause of tooth loss, affecting chewing function and threatening overall health [1-3].

The periodontal ligament stem cells (PDLSCs) can be obtained from the periodontal membrane of human third molars [4]. PDLSCs have self-renewal and multi-directional differentiation abilities and are considered potential “seed cells” for alveolar bone regeneration [5]. PDLSCs can differentiate into osteoblasts, chondrocytes, and adipocytes. The activity and multi-directional differentiation ability of mesenchymal stem cells (MSCs) were regulated by different pathways, such as nuclear factor-κB (NF-κB), PI3K/Akt, Wnt, *etc.* NF-κB plays a crucial role in the homeostasis of the immune system and inflammatory responses [6]. However, the local inflammatory microenvironment of the periodontium inhibits the regenerative ability of PDLSCs, resulting in periodontal tissue destruction.

Oxidative stress is found to be a key factor leading to periodontal tissue damage [7-9]. The imbalance in reactive oxygen species (ROS) and antioxidant factors leads to oxidative stress, resulting in pathological damage to periodontal tissue [10, 11]. ROS are oxygen-containing free radicals and derivatives related to oxygen metabolism, mainly derived from the oxidative phosphorylation reaction of mitochondria, namely cellular respiration; ROS mainly includes hydrogen peroxide radicals (HO_2_), hydrogen peroxide (H_2_O_2_), hydroxyl radicals (OH), singlet oxygen (O_2_), ozone (O_3_), and lipid peroxidation products [12, 13]. Corresponding to oxidase systems such as nicotinamide adenine dinucleotide phosphate (NADPH), other antioxidant systems that neutralize ROS production are superoxide dismutase, catalase (CAT), and glutathione [14, 15]. The physiological level of ROS is crucial for biological processes such as intracellular signal transduction and gene expression regulation. TNF-α leads to the production of ROS, and increased levels of ROS downregulate osteogenic differentiation markers osteocalcin, osteoblast-specific transcription factor, runt-related transcription factor 2 (Runx2), which significantly inhibits the MSCs differentiation into osteoblasts, reducing bone formation, promoting osteoclast formation, and disrupting normal cell function, even promoting cell apoptosis that leads to periodontal tissue damage and alveolar bone resorption [16, 17], Therefore, antioxidant stress should be included in periodontal treatment strategies based on PDLSCs’ osteogenic differentiation and protection of PDLSCs from ROS damage.

Melatonin (MEL) hormone is mainly produced by the pineal gland, which has anti-inflammatory, antioxidant, and immune regulatory properties [18, 19]. In terms of antioxidant activity, MEL and its metabolites constitute an efficient scavenger that can inactivate various reactive oxygen species under different conditions and eliminate free radicals through cascade action [20]. Yang *et al.*[21] found that MEL has a protective effect on cell apoptosis and oxidative stress. Arioz *et al.*[22] found that MEL can reduce the intracellular ROS production through mitochondria. MEL also plays an important antioxidant role in various diseases, including liver disease [23], hemolytic diseases [24], *etc.* Regarding anti-inflammatory effects, Kermeoğlu *et al.*[25] found that MEL significantly reduced the concentration of TNF-α, IL-1β, MMP-1, and MMP-2, exerting anti-inflammatory effects. MEL also plays an important role in osteogenic differentiation of bone marrow MSCs and downregulates osteoclast generation through the RANKL paracrine pathway [26]. In addition, MEL maintains the cellular function of MSCs by reducing cell aging, promoting cell migration, and inhibiting the production of ROS [27]. However, only a few studies have investigated whether MEL can reduce ROS levels and retain the osteogenic potential of hPDLSCs in the presence of TNF-α.

The purpose of this study was to evaluate MEL as an antioxidant in the osteogenesis process of hPDLSCs for the application in the bone regeneration of PDLSCs. Furthermore, we investigated whether MEL could protect the osteogenic differentiation ability of hPDLSCs and reduce ROS production in the presence of the inflammatory cytokine TNF-α.

## 2 MATERIALS AND METHODS

### 2.1 Materials

Antibodies to runt-related transcription factor-2 (RUNX2), NADPH oxidase1 (NOX1), osteopontin (OPN), and CAT were acquired from Abcam, Cambridge, UK. NF-κB pathway proteins (p65, phosphorylated p65, and IκBα), along with goat anti-mouse IgG and anti-rabbit IgG antibodies, were supplied by Cell Signaling Technology Inc., Boston, MA, USA. Antibodies against GAPDH were obtained from Proteintech, Rosemont, IL, USA. MEL was obtained from Sigma-Aldrich, St. Louis, MO, USA, and human TNF-α was sourced from Invitrogen, CA, USA.

### 2.2 Isolation and Characterization of hPDLSCs

This is an *in vitro* experimental laboratory study using human periodontal ligament stem cells (hPDLSCs). hPDLSCs were collected from the premolars of 19 healthy adolescent donors, aged 14 to 22, who underwent subtractive orthodontic extraction at the Stomatological Hospital, Guizhou Medical University. Ethical approval was obtained from the Ethics Committee of the affiliated Stomatological Hospital at Guizhou Medical University (approval number: 2022-54). All study participants provided informed consent. The hPDLSCs were retrieved from freshly extracted teeth and enzymatically digested with 3 mg/mL collagenase type I (Sigma-Aldrich, St. Louis, USA) and 4 mg/mL dispase II (Roche, Mannheim, Germany) for 30 min at 37°C [4]. The cell suspension was seeded into a 25 cm^2^ culture flask and maintained in medium containing α-minimum essential medium (α-MEM) (HyClone, USA), 10% fetal bovine serum (FBS, Gibco, USA), and 100 U/mL penicillin-streptomycin (HyClone, USA) under standard conditions (37°C, 5% CO_2_). The experiments used cells from the fourth generation of PDLSCs. To confirm cell surface markers, the PDLSCs were stained with antibodies against CD90-APC, CD146-PE, and CD45-FITC (1:100, BD Pharmingen, USA), with PBS used as a negative control. Flow cytometry (Beckman Coulter, USA) was employed to analyze the expression of these markers.

### 2.3 ALP Staining and Alizarin Red Staining

PDLSCs were cultured in medium containing 5 mM β-glycerophosphate, 50 µg/mL ascorbic acid, and 100 nM dexamethasone, with medium changes every two days for osteogenic differentiation. After 7 and 14 days of incubation, ALP staining was done using an ALP staining kit (Beyotime Biotechnology, Beijing, China). ALP activity was quantified on day 7 following the manufacturer's instructions [28]. On day 21, the culture supernatant was removed, and then the cells were fixed with formaldehyde (4%) for 15 min at room temperature. Then, the cells were washed with PBS 3 times and stained with 2% Alizarin Red S solution (Thermo Fisher Scientific, USA) for 2 min, followed by another 3 PBS washes. Finally, the calcium deposition was assessed.

### 2.4 Assessment and Measurement of ROS

The generation of ROS in hPDLSCs was assessed using the fluorescent probe 2′,7′-dichlorodihydrofluorescein diacetate (DCFH-DA; Invitrogen, Waltham, MA, USA). For adherent cells, DCFH-DA was added to 10 μmol/L α-MEM, and cells were incubated in 6-well plates in the dark for 20 min at 37 °C. After incubation, the cells were rinsed with PBS 3 times, and fluorescent signals were captured using a Leica DMI4000B fluorescence microscope (Leica, Wetzlar, Germany). For suspension cells (2 × 10^5^; n = 3), the same concentration of DCFH-DA in α-MEM was used, and incubation was carried out under identical conditions. Following 3 PBS washes, fluorescence intensity was analyzed by flow cytometry (Beckman Coulter, CA, USA) at excitation/emission wavelengths of 488/525 nm. Data were calculated by FlowJo (Becton, Dickinson and Company, Franklin Lakes, NJ, USA).

### 2.5 Western Blot

Cells maintained in osteogenic induction medium were thoroughly lysed using RIPA buffer supplemented with PMSF (Beyotime Biotechnology, Beijing, China). The resulting lysates were centrifuged, and the supernatant containing total protein was collected. Proteins were quantified using the BCA assay kit. Following protein separation and transfer, PVDF membranes were blocked on a shaker with buffer for 2 hours at room temperature. Then, the membranes were incubated with antibodies overnight at 4 °C, and then incubated with antibodies for 2 hours at room temperature [29].

### 2.6 Reverse Transcription Quantitative PCR (RT-qPCR)

Total RNA was isolated from cells across various groups using RNAiso Plus reagent (TaKaRa, Dalian, China). Complementary DNA (cDNA) was synthesized using the PrimeScript RT Master Mix (TaKaRa). Then, quantitative real-time PCR (qPCR) was conducted on a LightCycler 480 system (Roche, Basel, Switzerland) using SYBR Green I Master Mix (Roche). The expressions of RUNX2, OPN, NOX1, and CAT were quantified, with GAPDH serving as the internal reference gene. For each sample, the cycle threshold (Ct) of each target gene was normalized to that of GAPDH to obtain the ΔCt. Finally, the mean ΔCt from triplicate reactions was calculated, and relative gene expression levels were determined using the 2^-ΔΔCt^ method. The primer sequences used are shown in Table **1**.

### 2.7 Statistical Analysis

Descriptive statistics were calculated. Statistical analysis was done using SPSS 25.0 statistical software with One-way ANOVA and the Post-Hoc Tukey’s test. A *p* < 0.05 was considered statistically significant.

## 3 RESULTS

### 3.1 Characterization of hPDLSCs

The identity of the cultured hPDLSCs was verified by assessing their morphology and surface marker expression. Five days after initial seeding, optical microscopy revealed that the cells exhibited a spindle-shaped morphology consistent with previously described characteristics of hPDLSCs [30] (Fig. **1A**, **B**). The flow cytometry presented that the hPDLSCs were positive for CD90 and CD146 but negative for hematopoietic markers CD45 (Fig. **2**), which is similar to the previous study [30]. Also, the hPDLSCs showed osteogenic and adipogenic differentiation (Fig. **3**), which is another typical characterization of hPDLSCs.

### 3.2 Effect of MEL and TNF-α on Osteogenesis of hPDLSCs

To evaluate the effects of MEL and TNF-α on osteogenic differentiation, hPDLSCs were exposed to TNF-α (10 ng/mL) and varying MEL concentrations (0, 0.1, 1, 10, 100, and 200 μM). On day 7, alkaline phosphatase (ALP) activity was assessed. MEL treatment resulted in a dose-dependent increase in ALP activity in hPDLSCs. Treatment of both MEL (l00 μM) and TNF-α (10 ng/ml) in hPDLSCs led to significantly increased ALP activity compared to the control ones, which indicated that MEL was able to rescue the TNF-α-induced impaired osteogenesis of hPDLSCs (Fig. **4**). hPDLSCs were treated with MEL (l00 μM) and TNF-α (10 ng/ml) during osteogenic induction. ALP staining and activity assays were conducted on day 7 and day 14 to study the effects of TNF-α and MEL on bone formation. TNF-α treatment resulted in a decrease in ALP staining and activity, and MEL was able to result in TNF-α-induced impaired osteogenesis of hPDLSCs (Figs. **5** and **6**).

After 21 days of induction under various treatment conditions, Alizarin Red staining was conducted to assess mineralized matrix formation in hPDLSC cultures. The results indicated that TNF-α treatment alone led to a noticeable reduction in calcium deposition, whereas co-treatment with MEl mitigated this inhibitory effect on mineralization (Fig. **7**).

RUNX2 and OPN, two key regulators of osteogenesis, were evaluated to further investigate the osteogenic status of hPDLSCs under TNF-α and MEL exposure. Both RT-qPCR and Western blot analyses revealed that TNF-α treatment resulted in a progressive downregulation of RUNX2 and OPN expression levels. Treatment of TNF-α and MEL induced a significant reversal increase of Runx2 and OPN mRNA and protein expression (Figs. **8** and **9**). The results show that MEL resulted in TNF-α-induced inhibition of hPDLSCs osteogenesis.

### 3.3 MEL Reduced TNF-α-Induced ROS during Osteogenesis

To assess whether MEL can attenuate TNF-α-induced ROS generation during the osteogenic differentiation of hPDLSCs, cells were treated with TNF-α (10 ng/mL) alone or in combination with MEL (100 μM). As shown in Fig. (**10A**), TNF-α treatment alone led to a marked increase in ROS level on days 7 and 14. However, the addition of MEL significantly reduced ROS levels during osteogenesis. Flow cytometry analysis further confirmed that TNF-α elevated ROS production compared to the control group (Fig. **10B**), while co-treatment with MEL effectively suppressed this increase. These findings suggest that MEL mitigates TNF-α-induced oxidative stress during the osteogenic differentiation of hPDLSCs.

### 3.4 MEL Inhibited the Oxidative Enzyme and Improved the Antioxidative Enzyme during Osteogenesis

RT-qPCR and Western blot analyses showed that TNF-α significantly upregulated the expression of the oxidative enzyme NOX1, whereas co-treatment with MEL markedly suppressed NOX1 expression. Additionally, TNF-α decreased the expression of the antioxidative CAT enzyme, while MEL treatment effectively restored and enhanced CAT expression levels (Figs. **11** and **12**). These findings indicate that MEL can downregulate oxidative enzyme expression and promote antioxidative responses during the osteogenic differentiation of hPDLSCs under inflammatory conditions.

### 3.5 MEL Inhibited TNF-α-Induced NF-κB Signaling Activation during Osteogenesis

Western blot analysis showed that TNF-α alone markedly increased the phosphorylation of p65 and promoted the degradation of IκBα on days 7 and 14. However, co-treatment with MEL effectively attenuated these effects (Fig. **13**). These findings signify that MEL suppresses the NF-κB signaling pathway induced by TNF-α during osteogenic differentiation.

## 4 DISCUSSION

PDLSCs are a potential seed for alveolar bone regeneration, and their osteogenic differentiation is the biological basis for alveolar bone regeneration [5]. TNF-α plays a dominant role in periodontitis, promoting the absorption of alveolar bone by increasing the formation and activity of osteoclasts, and has a negative impact on the osteogenic differentiation of PDLSCs [31]. TNF-α-induced ROS production has been shown to significantly impair the osteogenic differentiation of PDLSCs, contributing to disrupted bone remodeling. In this study, we demonstrated that MEL effectively reduces ROS levels and preserves the osteogenic potential of PDLSCs in an inflammatory environment induced by TNF-α. These findings highlight the antioxidant role of MEL during osteogenesis and offer new support for its use in maintaining the regenerative capacity of PDLSCs. This study suggests that MEL holds potential as a therapeutic candidate for preventing bone loss associated with periodontitis.

In recent years, research has found that reactive oxygen species play a core role in the progression of many inflammatory diseases. The inflammatory microenvironment formation in periodontal disease increases the production and accumulation of ROS, while the body's antioxidant defense system is imbalanced. Excessive ROS causes oxidative stress, promotes the formation of osteoclasts, leads to the absorption of alveolar bone, and aggravates the damage to periodontal tissue [6, 16, 17]. The inflammatory cytokine TNF-α can activate NF-κB signal transduction and increase the expression of NOX, thereby inducing the production of ROS [32]. PDLSCs play a vital role in alveolar bone regeneration, and high levels of ROS can impair the self-renewal and osteogenic differentiation ability of hPDLSCs, which can impair tissue regeneration [33]. Therefore, exploring methods to reduce ROS levels is of great significance in protecting PDLSCs from oxidative damage.

Osteopontin (OPN) is a secreted phosphoprotein widely seen in the extracellular matrix of bone cells, and it has an important role in proliferation, migration, and adhesion of various bone-related cells, and plays a vital role in bone metabolism and balance [34]. In this study, TNF-α can reduce ALP activity and calcium nodules produced during osteogenic differentiation of hPDLSCs and downregulate the expression levels of osteogenic genes and proteins (including RUNX2 and OPN). After MEL treatment, the inhibitory effect of TNF-α was reversed, offsetting the interference of TNF-α on the osteogenic effect of hPDLSCs.

MEL is an endogenous neurohormone mainly secreted by the pineal gland, which can play a role in anti-inflammation, antioxidant function, immune regulation, cellular protection, and bone balance regulation [18, 19]. Studies have reported that MEL has an important role in the differentiation of MSCs, which are important for bone formation and mineralization. Jiang *et al.* [35] found that 100μM of MEL effectively promoted bone formation in MSCs through the translocation of Smad1/5/8 from the cytoplasm to the nucleus. In this study, we induced osteogenic differentiation of hPDLSCs and found that 100μM of MEL can maximize the differentiation of hPDLSCs inhibited by TNF-α. ALP is expressed in mineralized tissue cells and can increase the local rate of inorganic phosphates. The higher the ALP activity, the stronger the ability to promote mineralization, which plays a vital role in the hard tissue formation [36]. Alizarin red can stain the calcium matrix, and the degree of staining indicates the deposition of calcium ions in the extracellular matrix [37]. RUNX2 is the main transcription factor for osteoblast differentiation. It is a marker of osteoblast differentiation that helps in bone formation and reconstruction [38].

In addition, MEL is a strong natural antioxidant, which not only directly scavenges oxygen-free radicals but also regulates the balance between intracellular oxidation and antioxidant enzyme systems to exert antioxidant effects [20]. MEL can decrease the intracellular concentrations of ROS, superoxide, and H_2_O_2_, thus enhancing cell antioxidant capacity in bone marrow MSCs derived from osteoporotic rats [39]. In this study, we observed that TNF-α significantly increased the ROS levels of PDLSCs, while MEL reduced ROS generation, indicating that MEL can clear TNF-α-induced ROS. We then investigated the mechanism of MEL clearing ROS. The production and elimination of ROS mainly depend on the balance between oxidation and antioxidant enzyme systems. Excessive ROS production is usually due to the excessive activation of nicotinamide adenine dinucleotide phosphate (NADPH) oxidase. NOX1 is a key enzyme for redox signaling and belongs to the NADPH oxidase category [40]. Catalase (CAT) is an antioxidant enzyme that can catalyze H_2_O_2_ into oxygen and water, protecting cells from excessive ROS damage caused by endogenous metabolism or the external microenvironment [41]. This study found that TNF-α increased the gene expression significantly and protein expression of oxidase NOX1, while in contrast, the gene and protein expression of antioxidant enzyme CAT were significantly downregulated, which shows an imbalance between the oxidase and antioxidant enzyme systems (during ROS production in the presence of TNF-α). In the presence of TNF-α, MEL inhibited the expression of oxidase NOX1, improved the expression of antioxidant enzyme CAT, and cleared TNF-α-induced high-level ROS, thereby protecting hPDLSCs from oxidative damage during osteogenic differentiation and exerting strong antioxidant effects. Our results further supported the view that MEL has antioxidant effects.

The NF-κB signaling pathway is a prime regulatory factor in immune and inflammatory responses and is considered a major downstream target of TNF-α. The activation of NF-κB signaling adds to the TNF-α-inhibited osteogenesis of MSCs [42]. Moe *et al.*[43] mentioned that TNF-α is able to activate the NF-κB signaling and helps in the expression of NADPH oxidases, which results in high ROS levels. MEL can enhance the osteogenic differentiation ability of BMMSCs by inhibiting the MT2 receptor-NF-κB pathway [26], and it can also regulate oxidative stress and inhibit the production of nitric oxide (NO) through the NF-κB pathway [44]. In our study, we detected the expression and phosphorylation levels of NF-κB pathway proteins at different time points during the osteogenic differentiation process of hPDLSCs. The results showed that TNF-α treatment resulted in the activation of NF-κB pathway, which contributed to sustained high expression of NOX and high levels of ROS and resulted in the inhibition of the osteogenic differentiation ability of hPDLSCs. Therefore, MEL may exert its antioxidant and anti-inflammatory effects partially through the inactivation of the NF-κB pathway.

This study has some limitations. Firstly, only one inflammatory cytokine, TNF-α, was used in the production of ROS and to impair the osteogenic differentiation of hPDLSCs. More research can be done to study whether MEL can antagonize other inflammatory cytokines, such as IL-1 and IL-6, that induce inflammatory damage and oxidative stress. Secondly, we found that MEL can significantly inhibit NF-κB signaling activity induced by TNF-α. However, the role and potential mechanisms of MEL in NF-κB signaling in the inflammatory micro-environment still require further research. Finally, our research still lacks animal model experiments. Animal models of periodontitis will be constructed in further studies to provide more *in vivo* evidence for the osteogenic and antioxidant effects of MEL on hPDLSCs in an inflammatory microenvironment.

Although the present findings highlight the antioxidative and osteogenic potential of melatonin in TNF-α-challenged hPDLSCs, further investigations are warranted to facilitate its clinical translation. Future studies should focus on validating these effects in *in vivo* models of periodontitis, particularly to assess melatonin’s impact on alveolar bone regeneration, periodontal attachment preservation, and long-term tissue remodeling. Additionally, dose-response analyses and controlled delivery strategies—such as incorporation of melatonin into biomaterials, hydrogels, or scaffold-based regenerative systems—may optimize its therapeutic efficacy and local bioavailability.

At the molecular level, further elucidation of the crosstalk between oxidative stress pathways, inflammatory signaling, and osteogenic differentiation will help refine the understanding of melatonin’s regulatory mechanisms. Investigating its interaction with other pro-inflammatory cytokines and signaling axes relevant to periodontal disease may broaden its applicability. Ultimately, well-designed preclinical and clinical studies are essential to determine the safety, optimal dosing, and therapeutic potential of melatonin as an adjunctive treatment in periodontal regenerative therapy.

## CONCLUSION

This study demonstrates that melatonin effectively attenuates TNF-α-induced oxidative stress and restores the osteogenic differentiation capacity of human periodontal ligament stem cells in an inflammatory microenvironment. Melatonin reduced intracellular reactive oxygen species levels, preserved alkaline phosphatase activity, and enhanced the expression of osteogenic markers, including Runx2 and osteopontin. These effects were associated with suppression of NF-κB signaling, as evidenced by reduced p65 phosphorylation and stabilization of IκBα. Collectively, the findings provide mechanistic insight into the antioxidant and anti-inflammatory actions of melatonin and support its potential role in preserving periodontal stem cell-mediated osteogenesis under inflammatory conditions.

Despite the promising findings, several limitations should be considered. First, this study was conducted in an *in vitro* cell culture model using hPDLSCs, which may not fully replicate the complex biological environment of periodontal tissues *in vivo*, where multiple cell types, immune responses, and microbial factors interact. Second, the inflammatory condition was simulated using a single cytokine, TNF-α, whereas periodontitis involves a broader network of inflammatory mediators that may influence stem cell behavior. Third, the study focused primarily on the NF-κB signaling pathway; however, other signaling pathways related to oxidative stress and osteogenic regulation may also contribute to melatonin’s protective effects and require further investigation. Additionally, only limited concentrations and exposure times of melatonin were evaluated, leaving the optimal therapeutic dose and treatment duration uncertain. Finally, donor variability in hPDLSCs and the absence of long-term functional or *in vivo* validation may limit the generalizability of the findings. Future studies incorporating animal models, multi-cytokine inflammatory conditions, and clinical investigations will be essential to confirm the therapeutic potential of melatonin in periodontal regeneration.

## Figures and Tables

**Fig. (1):**
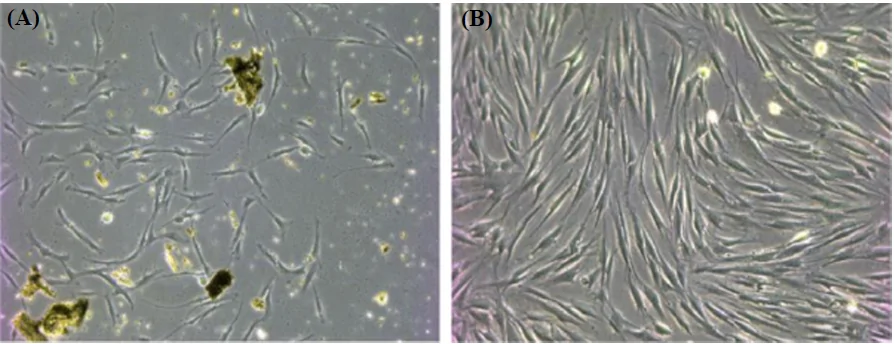
(**A**, **B**) Morphology observation of human periodontal ligament stem cells (100×).

**Fig. (2):**
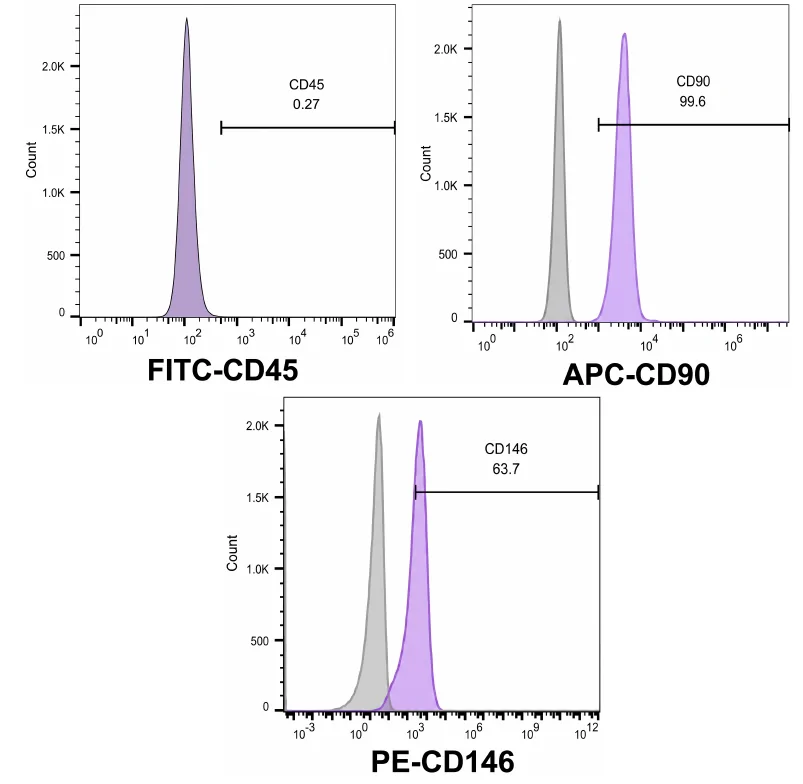
Detection of surface markers of human periodontal ligament stem cells.

**Fig. (3):**
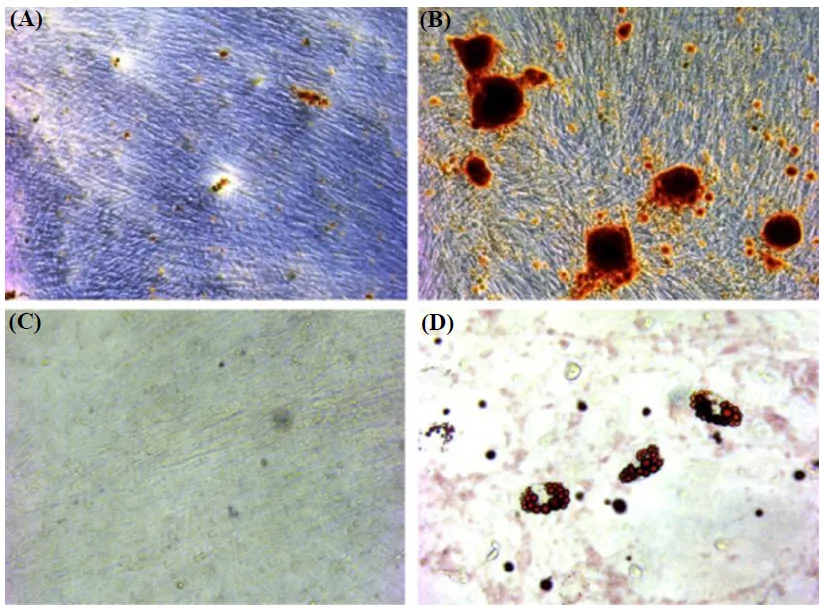
Detection of multidirectional differentiation ability of human periodontal ligament stem cells: (**A**) Alizarin red staining of negative control; (**B**) hPDLSCs showed osteogenic differentiation by Alizarin red staining analysis; (**C**) Oil-red-O staining analysis of negative control; (**D**) hPDLSCs showed adipogenic differentiation by Oil-red-O staining analysis.

**Fig. (4):**
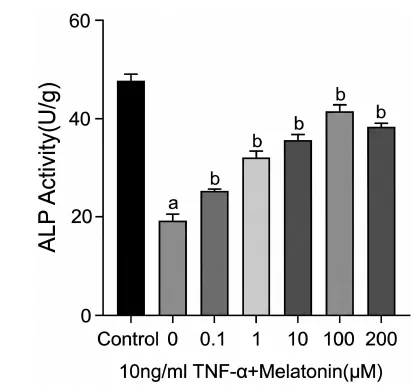
Alkaline phosphatase activity of hPDLSCs treated with TNF-α and melatonin at different doses. ^a^ *p*<0.05 compared with the control group; ^b^ *p*<0.05 compared with the 0μM group.

**Fig. (5):**
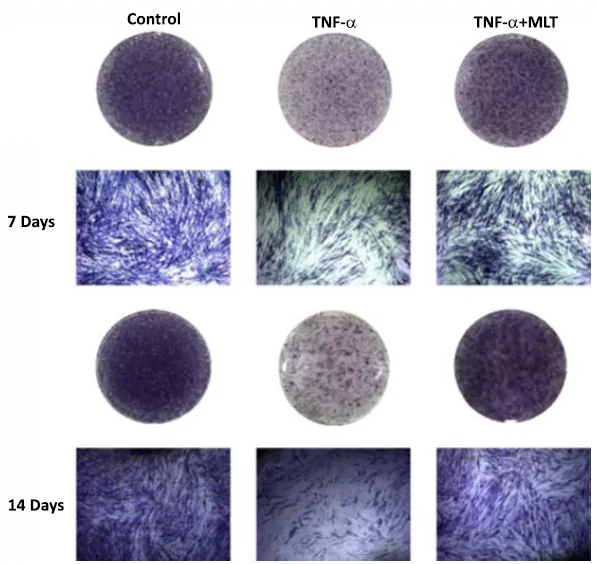
Alkaline phosphatase staining of human periodontal ligament stem cells in each group.

**Fig. (6):**
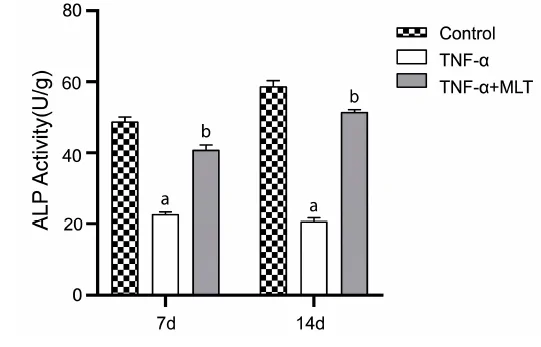
Alkaline phosphatase activity of human periodontal ligament stem cells in each group. ^a^ *p*<0.05 compared with the control group; ^b^ *p*<0.05 compared with the TNF-α group.

**Fig. (7):**
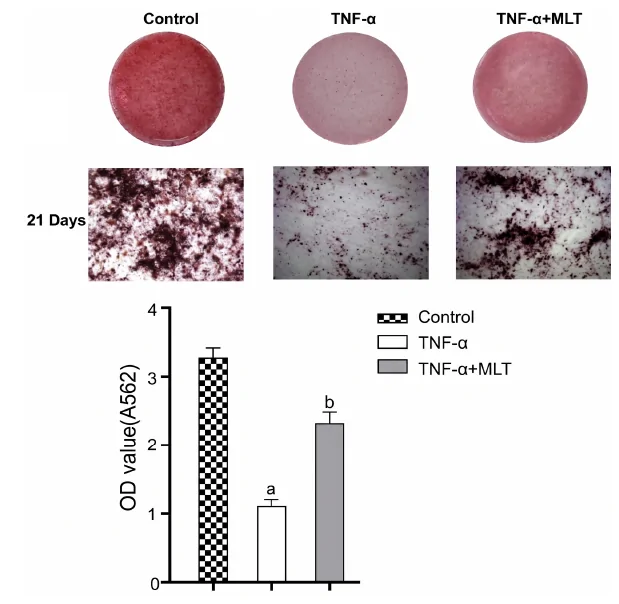
Alizarin red staining and semi-quantitative analysis of mineralized nodules. ^a^ *p*<0.05 compared with the control group; ^b^ *p*<0.05 compared with the TNF-α group.

**Fig. (8):**
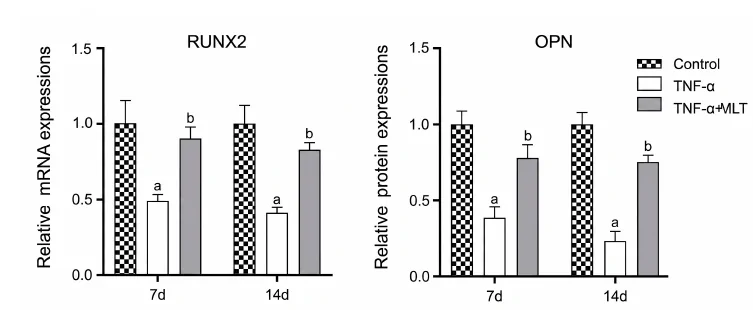
Expression of Osteogenesis-related genes in Human Periodontal Ligament Stem Cells in Various Groups. ^a^ *p*<0.05 compared with the control group; ^b^ *p*<0.05 compared with the TNF-α group.

**Fig. (9):**
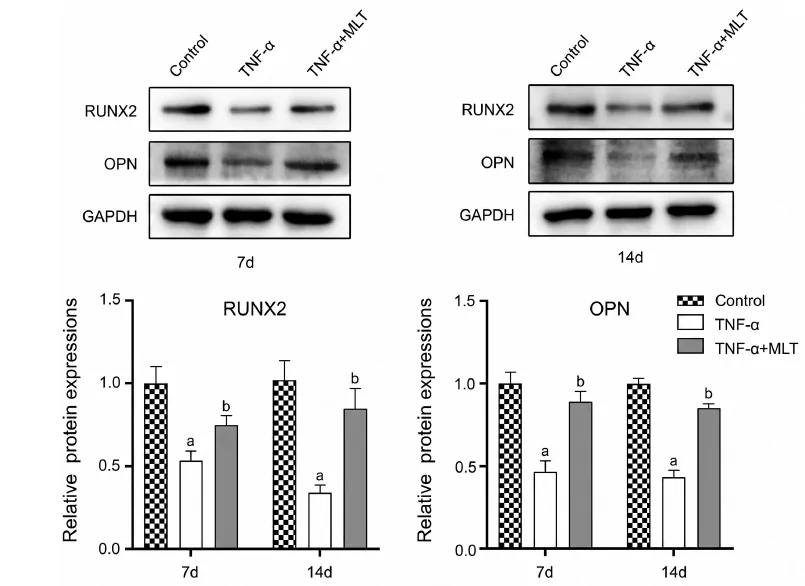
Expression of Osteogenesis-related Proteins in Human Periodontal Ligament Stem Cells in Various Groups. ^a^ *p*<0.05 compared with the control group; ^b^ *p*<0.05 compared with the TNF-α group.

**Fig. (10):**
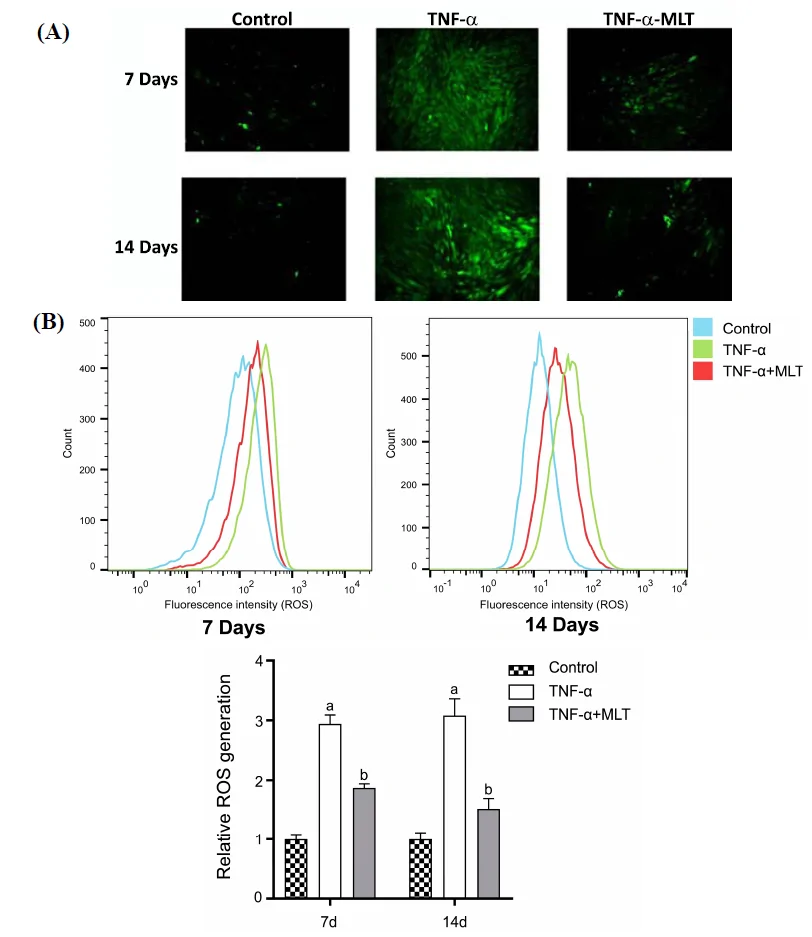
(**A**, **B**) The levels of ROS in human periodontal ligament stem cells in each group. ^a^ *p*<0.05 compared with the control group; ^b^ *p*<0.05 compared with the TNF-α group.

**Fig. (11):**
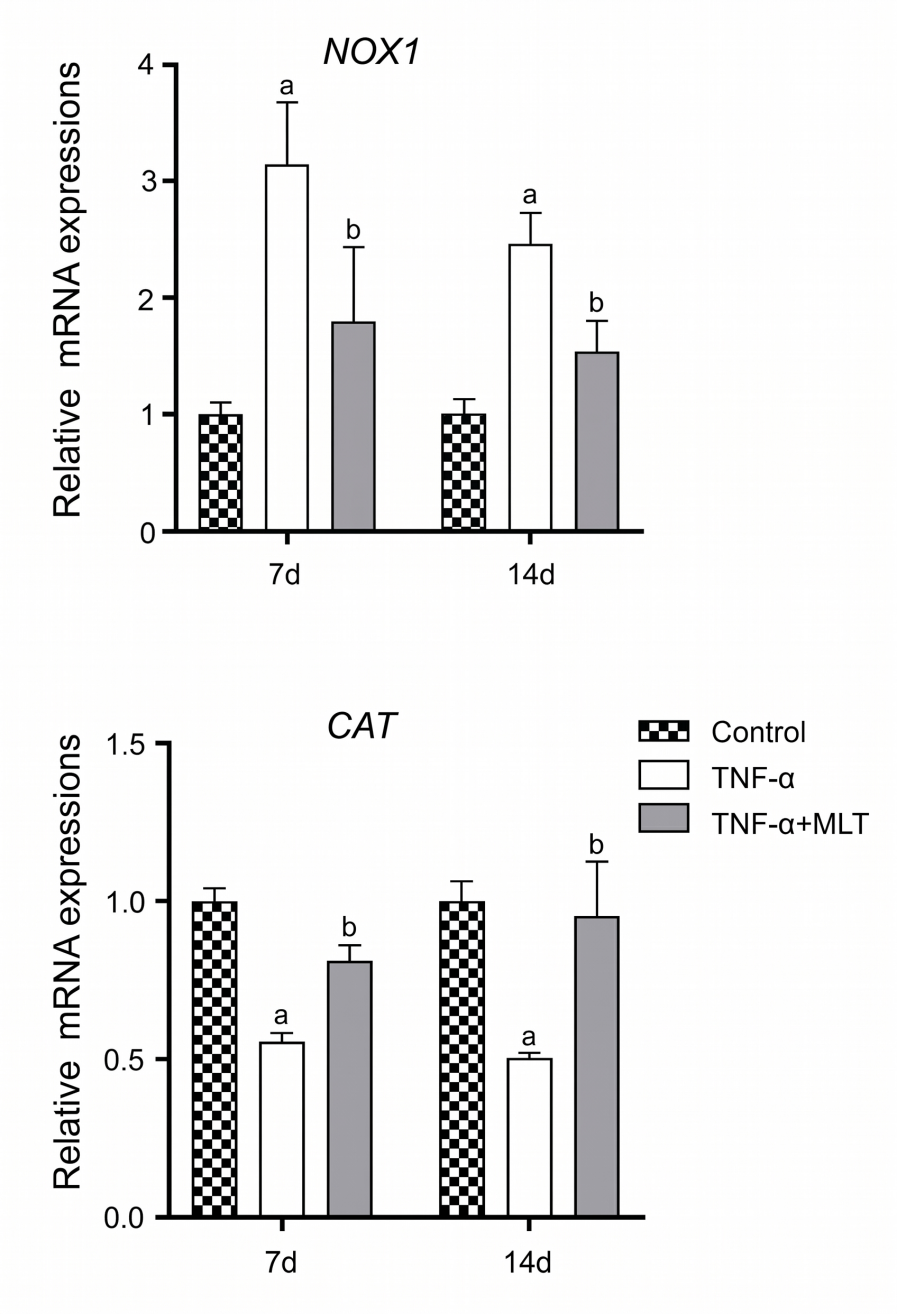
Expression of Osteogenesis-related genes in Human Periodontal Ligament Stem Cells in Various Groups. ^a^ *p*<0.05 compared with the control group; ^b^ *p*<0.05 compared with the TNF-α group.

**Fig. (12):**
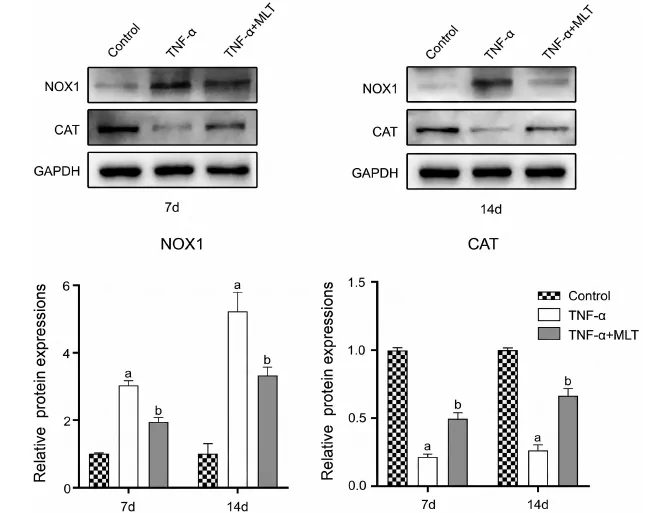
Expression of Osteogenesis-related Proteins in Human Periodontal Ligament Stem Cells in Various Groups. ^a^ *p*<0.05 compared with the control group; ^b^ *p*<0.05 compared with the TNF-α group.

**Fig. (13):**
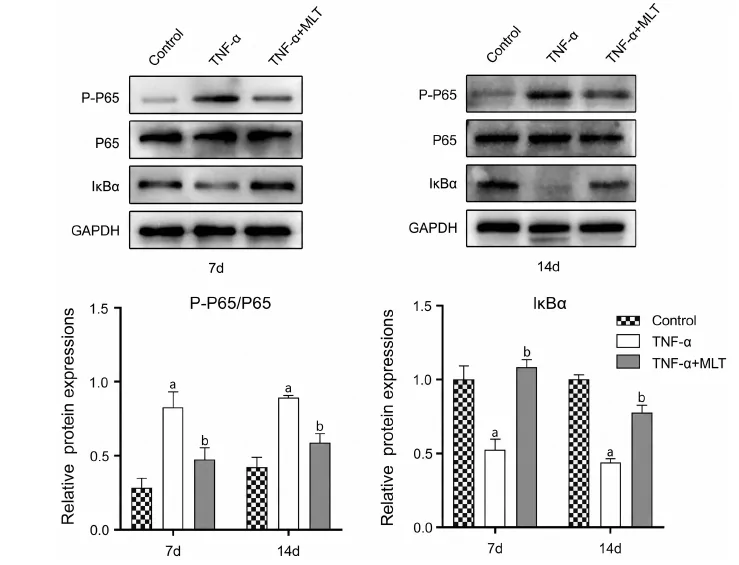
Melatonin inhibits TNF-α-induced activation of NF-κB signaling. ^a^ *p*<0.05 compared with the control group; ^b^ *p*<0.05 compared with the TNF-α group.

**Table 1:** The primer sequence used in this study.

**Gene**	**Primer Sequence**
GAPDH	Forward:5'- -3'CAGGAGGCATTGCTGCTGAT
	Reverse: 5'- -3' GAAGGCTGGGGCTCATTT
OPN	Forward:5'--3' CCAGTTGTCCCCACAGTAGAC
	Reverse: 5'- -3' GTCCTCGTCTGTAGCATCAGG
RUNX2	Forward:5'--3' AGGCAGTTCCCAAGCATTTCATCC
	Reverse: 5'--3'TGGCAGGTAGGTGTGGTAGTGAG
NOX1	Forward:5'--3' CGCACACTGAGAAAGCAA
	Reverse: 5'--3' CTGGAGAGAATGGAGGCA
CAT	Forward:5'--3' GAGCACAGCATCCAATATTCTG
	Reverse: 5'--3' CTCATTCAGCACGTTCACATAG

## Data Availability

The data presented in this study are available on request from the corresponding authors.

## STANDARDS OF REPORTING

STROBE guidelines were followed.
